# Do Big 5 Personality Characteristics and Narcissism Predict Engagement in Leader Development?

**DOI:** 10.3389/fpsyg.2018.01817

**Published:** 2018-09-28

**Authors:** Carrie A. Blair, Rachele E. Palmieri, Carmen Paz-Aparicio

**Affiliations:** ^1^Department of Management and Marketing, College of Charleston, Charleston, SC, United States; ^2^Institute of Entrepreneurship and Family Business, Universidad Carlos III de Madrid, Madrid, Spain

**Keywords:** personality, narcissism, leadership development, extraversion, agreeableness, Big 5

## Abstract

This study examines personality as a predictor of engagement behavior displayed during leader development programs. Leader development engagement behavior (LDEB) is measured by collecting self- and director ratings of behaviors displayed by undergraduate students during 1-year leader development programs (e.g., showing interest in a variety of topics, maintaining a positive attitude, arriving prepared for meetings, engaging with peers, and reflecting on development). Results suggest that factors of the Big 5 personality characteristics and the Narcissism Personality Inventory predict engagement behaviors in leader development. *Post hoc* analysis was conducted to better understand patterns of relationships between Big 5 factors and narcissism with specific LDEBs. Narcissism is negatively correlated with director ratings of reflection on development and engagement with peers. As a result of this research, leader development program directors should consider the benefits and risks of including narcissistic individuals in leader development programs.

## Introduction

### Personality Predicts Engagement in Leader Development

Organizations are increasing investment in leader development. In 2012, companies in the United States reported spending approximately $14 billion dollars annually on leader development (Leow and O’Leonard, 2012), and the amount spent on leader development increased 14% in 2013 (Schwartz et al., 2014). As the environment that surrounds organizations becomes increasingly complex, the expectation is raised that, “…all organizational members need to be leaders and all leaders need to be better prepared to participate in leadership” (p. 4; Day and Halpin, 2004).

When approximately 500 business executives were asked to rank their top human-capital concerns, around two-thirds of the executives chose leader development as their top priority (The Conference Board and McKinsey, 2012). Furthermore, the majority of Americans polled believe there is a leadership crisis (Rosenthal, 2012). In a theoretical piece summarizing what consulting psychologists can do to improve the state of leadership development, Kaiser and Curphy (2013) caution that despite significant increases in leader development spending, there is decreased confidence that leaders are prepared for the challenges that face organizations. Part of the issue is that leader development is a complex process, and its outcomes are difficult to measure. Specifically, leader development involves changes in the participants’ decision-making processes, perceptions of people and situations, understanding of organizational and environmental complexity, and interpersonal styles in order to make them better prepared to engage in leadership. There are different methods of leader development, including self-reflection, feedback, mentoring, role-modeling, and stretch goal assignments (McCauley, 2008). More research is needed to better understand leader development (Riggio, 2008; Day et al., 2014) and leader development assessment (Hannum and Craig, 2010).

Past leader development research has typically examined changes in job performance or behavioral outcomes following intervention (for a summary, see Hannum and Craig, 2010). In a recent review of the leader development literature, Day and colleagues note the limitations of using job performance as a leader development outcome. The authors note, “…the appropriate criterion for evaluation efforts is development and its markers rather than performance *per se*” (Day et al., 2014, p. 14). Day et al. (2014) also note the need to understand individual difference factors that predict development during leader development.

The aim of this study is to better understand personality as a predictor of leader development engagement behavior (LDEB). Big 5 personality characteristics (extraversion, agreeableness, conscientiousness, emotional stability, and intellectual imagination; Goldberg, 1992) and narcissism (Raskin and Terry, 1988; Gentile et al., 2013) are included as personality indicators. LDEBs are also examined *via* self- and supervisor ratings of actual behaviors displayed during leader development. The behaviors measured include factors important for self-development (Boyce et al., 2010) and cohort development (Galli and Müller-Stewens, 2012).

### Leader Development

There is a noteworthy difference between management development, leader development, and leadership development. Management development is a process that emphasizes “individual-based knowledge, skills, and abilities associated with formal management roles” (Day, 2000, p. 582). While training often prepares an individual for a specific role as part of a specific job, leader development addresses individual perceptions, motivations, and awareness in order to prepare the individual for complex jobs that include unpredictable roles and challenges. A meta-analysis of over a decade of research in leader development suggests that leader development tends to have a positive impact on outcomes (Collins and Holton, 2004).

Leader development can be made up of any of a number of components (Day, 2000; Day and Halpin, 2004; McCauley, 2008). For example, any of the following individually or in some combination is often considered leader development:

1. multisource feedback in which participants build intrapersonal competence through increased self-knowledge and self-awareness of one’s impact on others;
2. executive coaching which focuses on the assessment, challenge, and support of an individual;
3. mentoring between leaders of different experience levels who share a relationship to develop intrapersonal competence and a strategic perspective on personal and organizational development;
4. networking to develop social capital and awareness of sources of support within the organization;
5. job assignments that place leaders in situations of unfamiliar challenges and responsibilities in order to teach and develop the leader through first-hand experience; and
6. action learning driven by business imperatives with emphasis on challenge and reflection.

Leaders develop individually (Day, 2000), but leadership development programs (LDPs) commonly involve multiple leaders participating as a cohort throughout the leader development process. The distinction between leader development and leadership development is important. That is, leader development often occurs within the context of a larger organizational initiative toward leadership development, which focuses on developing multiple people for future roles and better decision-making (Day et al., 2014). For many LDP programs, the job assignments, feedback, and action learning occur in the context of a group of individuals brought together in order to not only develop individually but also to learn about other groups within the organization, recognize opportunities for innovation, and increase communication (Day et al., 2014). Thus what happens among a group of people enrolled in an LDP could be important for the overall development of social capital to occur as a result of leadership development (Galli and Müller-Stewens, 2012).

Recent research has examined the mechanisms that lead to successful leadership development within firms. For example, recent research includes an examination of the factors associated with individuals who engage in self-development for the purpose of improving their leadership skills (Boyce et al., 2010). As a second example, leader development has long been associated with the development of human capital within a firm. More recent research has also shown that leadership development has impact on the development of social capital within a firm (Galli and Müller-Stewens, 2012). Here, social capital is defined as “the quality created between people, whereas human capital is a quality of individuals” (p. 178). As a result, the quality of the cohort can be a driver of the success of the other elements of the LDP.

Along a similar vein, the focus of the current study is to examine personality predictors of LDEBs displayed during the course of leader development. These include factors that are thought to contribute to self-development and cohort development during the course of an LDP.

### Personality in the Context of Leader Development

In studies that have examined the effect of multiple factors on the behavior of individuals participating in leader development, personality consistently emerges as an important predictor of leader development participation and the general emergence of leadership (Noe and Wilk, 1993; Maurer and Tarulli, 1994; Funderburg and Levy, 1997; Maurer et al., 2002; Strang and Kuhnert, 2009; Hannah and Avolio, 2010; Day et al., 2012, 2014). However, less research has examined the relationship between personality and LDEBs. The relationship between personality and LDEBs is explored further in the following sections.

#### Big 5 Personality Characteristics

Personality characteristics predict behavior across a variety of settings. The five-factor model of personality is commonly used in personality research (Goldberg, 1990). Measures of the five-factor model have demonstrated stability over time (Costa and McCrae, 1988; Digman, 1989), and the five-factor model has consistently demonstrated correlation with general job performance (Barrick and Mount, 1991). In a meta-analytic review of the research, the five-factor characteristics extraversion, intellectual curiosity (or openness to experience), emotional stability, and conscientiousness were also associated with leadership effectiveness and leadership emergence (Judge et al., 2002), making these traits particularly relevant traits to consider in relation to behaviors displayed during leader development.

Still, while the Big 5 has been historically linked with leader behavior, the purpose of the current research is to better understand LDEBs. Various factors of the Big 5 show a pattern of relationships with behaviors potentially displayed during LDPs. That is, conscientiousness, openness to experience, and emotional stability have shown a positive relationship with coaching interventions (Stewart et al., 2008) and extraversion, conscientiousness, and openness to experience have shown a positive relationship with transfer of training (Barrick and Mount, 1991). While agreeableness has not demonstrated such a consistent relationship with leadership outcomes in the past, agreeableness is consistently associated with behaviors important for teamwork and cohort development (Bell, 2007). Conscientiousness and openness to experience have also demonstrated a pattern of relationship with individual propensity to engage in self-development as a means to improve leadership skill (Boyce et al., 2010).

Considering these arguments, and the frequency with which Big 5 characteristics relate to performance outcomes across the literature, we propose the following hypothesis:

*Hypothesis 1*: Big 5 personality characteristics are positively related to leader development engagement behaviors.

### Narcissism

Narcissism is specifically examined in the context of this study as narcissism is often associated with leader emergence (Paulhus, 1998; Brunell et al., 2008; Judge et al., 2009; Wales et al., 2013) and leader derailment (Conger, 1990; Hogan and Hogan, 2001). Narcissistic personality is characterized as showing an overwhelming need for admiration and attention, lacking empathy, and feelings of grandiosity (American Psychiatric Association, 2013). Narcissists tend to be attracted to leadership positions (Kets de Vries and Miller, 1985; Kets de Vries, 2004), and it is likely that “the same characteristics that facilitates [a narcissistic] individual’s emergence as a leader can also make this person a potentially destructive leader” (Brunell et al., 2008; see also Maccoby, 2004).

While leader development is often prescribed as an intervention for the derailing narcissist (Harms et al., 2011), narcissistic tendencies make it unlikely that narcissists will effectively engage in leader development. For example, one hallmark of leader development is receiving and responding to feedback from various sources (Day et al., 2014). However, narcissists tend to be sensitive to criticism (Rhodewalt and Morf, 1998; American Psychiatric Association, 2013), and narcissism is associated with resistance to feedback (Kernis and Sun, 1994). Even in an assessment center where stakes were low and feedback was not personal, narcissism predicted a negative reaction to feedback (Blair et al., 2017).

A second important component of leader development is that it serves as an opportunity to develop social capital within the organization (Galli and Müller-Stewens, 2012). However, it is possible that a strong narcissistic personality in the program could hinder the developmental progress of other program participants. In general, narcissists tend to exploit others (Kets de Vries and Miller, 1985), react with rage when favors are not reciprocated (Meier and Semmer, 2012), are threatened by the success of others (Lubit, 2002), and have a tendency to hoard knowledge that may make other people successful (Kearney, 2010). In the workplace, narcissism is associated with counterproductive work behaviors (Penney and Spector, 2002; Blair et al., 2017), and past research indicates a relationship between narcissism and supervisor ratings of interpersonal performance and integrity (Blair et al., 2008). Research examining the relationship between narcissism and group membership indicates that group members might initially see narcissistic team members as agreeable and competent but later reject these same team members due to arrogance and other negative behaviors (Paulhus, 1998).

Although there is little research examining the relationship between narcissism and leader development outcomes, there is at least some evidence that narcissism and similar tendencies decrease the effectiveness of leader development. Harms et al. (2011) examined the impact of several “dark side” traits, including narcissism, on leader development outcomes. Importantly, in their research, they expected to find that traits such as narcissism would reduce the effectiveness of leader development interventions. Instead, they found that traits associated with narcissism were positively associated with some leader development outcomes. Like most leader development research (see Hannum and Craig, 2010), Harms et al. (2011) study examined performance outcomes following leader development. However, in a summative piece on the leader development literature, Day et al. (2014) note that job performance as an outcome is “contaminated (p. 77)” and “…is affected by many things other than leadership skills (p. 77).”

In the current research, we examine LDEBs rather than job performance. Because narcissism has shown negative correlation with similar behaviors to LDEBs, we propose the following hypothesis:

*Hypothesis 2*: Narcissism is negatively related to overall leader development engagement behaviors.

## Materials and Methods

### Participants

Approximately 467 students at a university in the Southeastern United States were solicited to participate in the study between 2013 and 2015. The majority of the participants were undergraduate students, and a small subset were graduate students enrolled in the university’s MBA program. Participants were selected based on their participation in at least one of the 1-year LDPs at the University. The common hallmarks across the programs were that they were designed to span the length of one academic year (August–May), encouraged leader development, provided opportunities for personal and professional growth, sought students who were leaders or driven members of the university’s community, and expected students to interact throughout challenging personal and professional growth opportunities. Twelve programs at the university met these qualifications. The LDPs represented programs across the university, including honors, business, education, and communication. The participants varied in academic interests and year within the university. Of the 467 participants, 134 responded (29% response rate). Of those, 114 also received director ratings of performance. Out of the participants, the majority were female (66.4%) and the average age was 21.46 years.

### Ethics Statement

This study was reviewed and approved by the College of Charleston Institutional Review Board with written informed consent from all participants. All participants gave written informed consent in accordance with the Declaration of Helsinki.

### Materials

The researchers set up preliminary meetings with each of the program’s directors to discuss how he or she would describe exemplary and engaged students’ behavior in each of the LDPs. During this meeting, they also discussed the director’s potential involvement in a study that measured the relationship between their students’ personality factors and performance within their program. The behaviors used to measure leader development engagement were determined based on information provided during these interviews. Each of the directors reviewed the items, and felt that they would be able to answer the questions about their students after engaging with the students for several months in the program. Both the students and directors were asked to complete this measure about the students’ behavior during the LDP. The researchers also determined which personality measure would accurately measure narcissism and could be embedded into the Big 5 to accompany the performance measure in the student evaluation.

The research was first conducted during the 2013–2014 academic year and then repeated during the 2014–2015 academic year. Toward the end of the academic year, the researchers sent each director the consent letter with the survey link embedded, and asked that they forward the email to their students, inviting participation. While the surveys were collected over the course of 2 years, the timeframe within each year for collecting the surveys stayed the same. That is, at the start of the second semester in the program, students were sent the student survey. Six weeks later (toward the end of the 1-year period of working with the students), the directors were sent surveys to complete for those students who completed the student survey. While the number of surveys completed by each director varied, the size of the leader development programs were relatively equivalent. That is, each program contained 10-20 students in a cohort assigned to the director, thus there was significant opportunity for interaction between each student and the director.

Student participants were entered into a raffle to receive one of several $20 gift cards; directors received a $5 coffee gift card for participating.

### Narcissism

The Narcissism Personality Inventory Short Scale (NPI-13) was used in this study (Gentile et al., 2013). The NPI-13 includes 13 forced choice pairs of attributes (see Cronbach’s alpha coefficient of reliability in **Table 1**). Scores can be evaluated as three subscales of narcissism. The first of the subscales is Leadership/Authority (α = 0.54, e.g., “I like having authority over other people”; “I am a born leader”). The second of the subscales is Grandiose/Exhibitionism (α = 0.67; “I like to look at my body”; “I will usually show off if I get the chance”). The third of the scales, Entitlement/Exploitativeness (α = 0.50), is described by Gentile et al. (2013) as the most maladaptive of the three scales (e.g., “I insist on getting the respect that is due me”; “I expect a great deal from other people”).

**Table 1:** Correlations for personality variables with overall performance ratings.

	Variable	1.	2.	3.	4.	5.	6.	7.	8.	9.	10.	11.
1.	NPI – total	(0.55)										
2.	NPI – Leadership/Authority	0.55^**^	(0.54)									
3.	NPI – Grandiose/Exhibitionism	0.67^**^	-0.04	(0.67)								
4.	NPI – Entitlement/Exploitativeness	0.66^**^	-0.25^**^	0.10	(0.50)							
5.	Extraversion	0.12	0.29^**^	0.02	-0.05	(0.74)						
6.	Agreeableness	-0.26^**^	-0.23^**^	0.07	-0.42^**^	0.68^**^	(0.76)					
7.	Conscientiousness	0.07	0.11	-0.03	0.08	0.64^**^	0.73^*^	(0.82)				
8.	Emotional stability	-0.11	-0.07	0.05	-0.22^**^	0.67^**^	0.72^**^	0.70^**^	(0.74)			
9.	Intellectual imagination	-0.08	0.07	-0.01	-0.22^**^	0.67^**^	0.85^**^	0.78^**^	0.74^**^	(0.68)		
10.	Total self-rating	-0.16^*^	-0.02	-0.07	-0.21^**^	0.17^*^	0.54^**^	0.04	0.23^**^	0.27^**^	(0.77)	
11	Total director rating	-0.21^*^	-0.19^*^	-0.09	-0.31^**^	0.03	0.09	-0.00	0.13	0.02	0.14	(0.92)
	Gender	-0.18^*^	-0.22^**^	-0.02	-0.15^*^	-0.16^*^	0.06	-0.14	-0.26^**^	-0.14^*^	0.06	-0.01
	Age	0.06	0.02	0.04	0.05	0.03	0.04	0.08	0.04	0.08	0.07	0.21^**^
	*N*	124	124	124	124	134	134	134	134	134	124	124
	Mean	5.22	1.47	2.73	1.02	3.04	3.75	3.57	2.86	3.60	4.85	4.97
	Standard deviation	2.15	0.98	1.38	1.04	1.14	1.20	1.21	1.07	1.14	0.68	0.82

*^∗^p < 0.05, ^∗∗^p < 0.01 one-tailed. Gender 1, male; 2, female. NPI (Narcissism Personality Inventory). Numbers in parentheses along the diagonal are reliability coefficients.*

### The Big 5 Personality Characteristics

Students completed the 50-Item Set of IPIP Big-5, which is a version of the Big 5 Personality Assessment (Goldberg, 1992) and answered a variety of demographic questions (see Cronbach’s alpha coefficient of reliability in **Table 1**). The Big 5 Personality Assessment measured the Big 5 factor markers including extraversion (α = 0.74), agreeableness (α = 0.76), conscientiousness (α = 0.82), emotional stability (α = 0.74), and intellect/imagination (α = 0.68). The measure consisted of 50 statements with 7-point Likert responses (very inaccurate, inaccurate, moderately inaccurate, neither inaccurate nor accurate, moderately accurate, accurate, and very accurate).

### Leader Development Engagement Behaviors (LDEBs)

In a summative piece on leader development research, Day et al. (2014) highlight limitations with research using job performance and changes over time as leader development outcomes. Day and colleagues note, “The field needs to focus on identifying and tracking appropriate markers or proxies for development that go beyond a fixation on rated job performance” (p. 77). In response to this call, the measure of LDEBs was developed through an inductive process *via* interviews with the program directors. The researchers had a discussion with each director to share ideas about effective behaviors of students engaged in leader development programs. The researchers encouraged the directors to think about the program’s objectives and determine how their ideal student would behave. The ideal student was broadly defined as someone who was engaged in the program’s opportunities to develop relationships with speakers and peers, to network, and to absorb the lessons in order to better themselves. Each program director used different terms to describe an effective set of behaviors, but the researchers identified areas of consistency and overlap in order to create a scale for LDEBs. Before conducting the research, the researchers asked the directors to confirm that the developed scale was an accurate depiction of effective behaviors.

While the scale was developed *via* an inductive process, the items included in the scale align with factors associated with leader development. For example, Boyce et al. (2010) defined leader self-development as “a process in which leaders take personal responsibility for initiating, sustaining, and evaluating growth in their own leadership capacities and in their conceptual frames about the conduct of leadership” (p.162). Likewise, items in the questionnaire used in the current study include questions pertaining to showing initiative to solve problems and interest in learning about different subjects. As a second example, Galli and Müller-Stewens (2012) expanded the leader development literature by focusing on social capital as a leadership development outcome within organizations. In building a case for leadership development as a means to develop social capital for the organization, Galli and Müller-Stewens (2012) cite Day’s (2000) definition of leadership development as “…building the capacity for groups of people to learn their way out of problems that could not have been predicted” (Day, 2000, p. 582) and focuses “on building networked relationships among individuals that enhance cooperation and resource exchange in creating organizational value” (Day, 2000, p. 585). Likewise, items in the LDEB scale developed for this study measure engagement with peers and taking initiative to solve problems.

The resulting six-statement questionnaire was created to be completed by both students (self-ratings) and directors (other-ratings) (see **Appendix A**). The results are reported based on overall self-ratings (α = 0.77) and overall director ratings (α = 0.92) on the six-item questionnaire. *Post hoc* analysis was also conducted examining the relationship between the personality factors and each of the LDEBs. Both questionnaires were based on 6-point scales (strongly disagree, disagree, slightly disagree, slightly agree, agree, or strongly agree).

The leader development programs used in this study occurred within the context of a university setting. Additional explanation is provided here to show how these behaviors might be exhibited in this context. For example, a student might receive a high rating on “shows interest” if she came equally prepared to all events, even those events that were less relevant to her career interests. For example, a participant from accounting would receive a high rating if she consistently showed interest in events focused on other areas, like marketing or supply chain management. A student would receive high rating on “positive attitude” if he maintained a good attitude even when events were dull or poorly executed. For “engaged with peers,” a student would receive a high rating if he consistently worked to get to know other participants on a personal level by planning trivia nights or coffee breaks. For the question “arrives prepared” a student would receive low rating if he consistently made statements indicating he had not prepared for the visit (e.g., by asking irrelevant questions). A student would receive low rating for “takes initiative” if he or she constantly came to the program administrators with problems or complaints, expecting program administrators to solve problems on her behalf. A student might receive low rating for “reflects on development” if she is offended by negative feedback shared during the course of the program.

Two additional questions were included in the director questionnaire to evaluate the behavior of each of their students (see **Appendix A**). These questions asked directors for their opinion about the student’s potential to be a successful leader and his/her potential to derail during his/her career.

In order to check the appropriateness of using these questions, we have conducted two separate Exploratory Factor Analyses (See **Supplementary Table S1**), with the aim of investigating the dimensionality of the measures (one for LDP Self Rating and another one for LDP Director Rating). The question values were subjected to principal components analysis (PCA). The Kaiser-Meyer-Olin value reached in both self- and director dimensions the recommended value of 0.6 (Kaiser, 1970, 1974), and Bartlett’s Test of Sphericity (Bartlett, 1954) reached statistical significance, supporting the factorability of the two different variables (LDP Self and Director Rating). Thus, the analysis supports the measurement of LDP based on the questions stated in the questionnaire.

### Demographic Variables

Students also answered three demographic questions regarding their gender, age, and race/ethnicity. Out of the whole sample, the majority were female (66.4%) and the average age was 21.46 years. We used these variables as control variables in our study based on previous literature (Strang and Kuhnert, 2009; Brailovskaia et al., 2017).

## Results

**Table 1** presents the means, standard deviations, sample size, and Pearson product moment correlations for the study variables. **Tables 2** and **3** present the results of the multiple linear regression analyses for testing our hypotheses, analyzing the effect of the Big 5 personality characteristics and narcissism beyond overall self-ratings and director ratings of LDEB, respectively. This analysis is used to explore and quantify the relationship between the dependent variable and several independent or predictor variables, as well as to develop a linear equation with a predictive objective.

**Table 2:** Results of the regression model (LDP Self Rating Total).

	Model 1			Model 2			Model 3		
	Coeff.	SE	Sig.	Coeff.	SE	Sig.	Coeff.	SE	Sig.
Constant	1.071	0.617	^∗^	4.688	0.391	^∗∗∗^	4.770	0.387	^∗∗∗^
Big 5 personality characteristics: extraversion	0.117	0.067	^∗^	-	-		-	-	
Agreeableness	0.657	0.104	^∗∗∗^	-	-		-	-	
Conscientiousness	0.063	0.077		-	-		-	-	
Emotional Stability	0.049	0.077		-	-		-	-	
Intellectual Imagination	0.153	0.104		-	-		-	-	
NPI-13 dimensions									
Grandiose/Exhibitionism (GE)	-	-		-0.024	0.044		-	-	
Entitlement/Exploitativeness (EE)	-	-		-0.138	0.061	^∗∗^	-	-
Leadership/Authority (LA)	-	-		0.025	0.065		-	-	
NPI-13 total score							-0.048	0.029	^∗^
Age	0.003	0.011		0.012	0.013	^∗∗∗^	0.012	0.013	
Gender	-0.193	0.122		0.053	0.130		0.049	0.129	
*R* square	0.364			0.056			0.032		
Adjusted *R* square	0.325			0.016			0.008		
*F* value	9.478^∗∗∗^			1.395			1.329		
No. of observations	124			124			124		

*(i) Significance at ^∗^p < 0.10, ^∗∗^p < 0.05, ^∗∗∗^p < 0.01; (ii) SE, standard error. Source: Authors dataset.*

**Table 3:** Results of the regression model (LDP Director Rating Total).

	Model 1			Model 2			Model 3		
	Coeff.	SE	Sig.	Coeff.	SE	Sig.	Coeff.	SE	Sig.
Constant	3.567	0.644	^∗∗∗^	4.227	0.633	^∗∗∗^	4.163	0.620	^∗∗∗^
Big 5 personality characteristics				-	-		-	-	
Extraversion	-0.035	0.092							
Agreeableness	0.170	0.133		-	-		-	-	
Conscientiousness	-0.120	0.100		-	-		-	-	
Emotional stability	0.214	0.116	^∗^	-	-		-	-	
Intellectual imagination	-0.176	0.138		-	-		-	-	
NPI-13 dimensions									
Grandiose/Exhibitionism (GE)	-	-		-0.056	0.053		-	-	
Entitlement/Exploitativeness (EE)	-	-		-0.083	0.075		-	-
Leadership/Authority (LA)	-	-		-0.143	0.082	^∗^	-	-	
NPI-13 total score							-0.085	0.035	^∗∗∗^
Age	0.063	0.024	^∗∗∗^	0.061	0.024	^∗∗∗^	0.063	0.024	^∗∗^
Gender	-0.015	0.176		-0.062	0.161		-0.042	0.159	
*R* square	0.104			0.107			0.100		
Adjusted *R* square	0.050			0.066			0.075		
*F* value	1.923^∗^			2.586^∗∗∗^			4.069^∗∗∗^		
No. of observations	124			114			114		

*(i) Significance at ^∗^p < 0.10, ^∗∗^p < 0.05, ^∗∗∗^p < 0.01; (ii) SE, standard error. Source: Authors dataset.*

Overall, the results offered partial support for the first hypothesis, that Big 5 personality characteristics would influence LDEBs (**Table 2**). Model 1 presents the results for the Big 5 personality factors and the control variables age and gender, considering the overall self-ratings of leader development behavior. In this model, the coefficient for agreeableness is significant and positively related to the self-report of LDEB (β = 0.657, *p* < 0.01). In this study, extraversion, conscientiousness, emotional stability, and intellectual imagination were not significantly correlated with self-ratings of overall LDEB.

Model 1 in **Table 3** presents the regression results between the Big 5 personality characteristics and director ratings of overall LDEB. In this model, the coefficient for emotional stability reached statistical significance (β = 0.214, *p* < 0.10) and also the coefficient for the control variable age (β = 0.063, *p* < 0.01). In this study, extraversion, agreeableness, conscientiousness, and intellectual imagination were not significantly correlated with director ratings of overall LDEB.

The results partially confirm the hypothesis that narcissism is negatively related to leader development behaviors. Analysis based on narcissism subscales lend further partial support to this hypothesis. Models 2 and 3 in **Tables 2** and **3** show the regression results for testing hypothesis 2. Model 2 in **Table 2** shows the potential effect of the narcissism subscales on self-rating of LDEBs. In this model, the coefficient for Narcissism-Exploitative/Entitlement reached statistical significance (β = -0.138, *p* < 0.05).

Model 2 in **Table 3** shows the negative and significant coefficient for Narcissism-Leadership/Authority (β = -0.143, *p* < 0.10) and the control variable age (β = 0.061, *p* < 0.05) on the director rating of LDEB. It is interesting to note that the subscale Narcissism-Grandiosity/Exhibitionism was neither significantly correlated with overall self-ratings or director ratings of LDEB.

Model 3 in **Tables 2** and **3** show the regression results for the total overall narcissism rating, not considering the narcissism subscales. Model 3 (**Table 2**) shows significant coefficient for total narcissism behavior over self-rating of LDEB (β = -0.048, *p* < 0.10). There is also support for the relationship between total narcissism behavior and director rating of LDEB (β = -0.085, *p* < 0.01). Also, the coefficient for the control variable age reached statistical significance (β = 0.063, *p* < 0.05). That is, total narcissism behavior was significantly and negatively correlated with overall director rating of LDEBs and also with overall self-rating of LDEBs.

### *Post hoc* Analysis

Given the relationships between individual difference factors and overall LDEBs, and given the relative dearth of research exploring LDEBs, further analyses were conducted to understand the mechanisms that underlie the relationships. That is, in their exploration of leadership development as a means to increase social capital within a firm, Galli and Müller-Stewens (2012) note that even though it is commonly understood that leadership development has a positive impact on organizational outcomes, it is generally misunderstood how this positive impact occurs (see also Avolio, 2005). Furthermore, Day et al. (2014) call for more focus on identifying “…appropriate markers or proxies of development” (p. 77). Thus, we also examined the relationship between narcissism and specific engagement behaviors displayed during the course of leader development. Thus, *post hoc* analyses were conducted in order to understand the relationship between the personality factors and specific leader development behaviors.

Correlations are presented in **Table 4**. Several of the Big 5 Factors were correlated with specific leader development behaviors, as expected. For example, conscientiousness was positively correlated with self-reports of arriving prepared (*r* = 0.23, *p* < 0.05), emotional stability was positively correlated with maintaining a positive attitude (*r* = 0.18, *p* < 0.05), and agreeableness was positive correlated with engagement with peers (*r* = 0.50, *p* < 0.01).

**Table 4:** Correlations for personality with leader development behaviors.

	Narcissism sub-scales				The Big 5 personality characteristics				
	Total	LA	GE	EE	E	A	C	ES	II
Self-ratings									
Shows interest	-0.25^**^	-0.11	-0.14	-0.22^**^	0.03	0.51^**^	-0.07	0.21^**^	0.19^*^
Positive attitude	-0.16^**^	0.04	-0.12	-0.21^*^	0.19^*^	0.59^**^	0.02	0.18^**^	0.12
Arrives prepared	0.02	0.12	-0.02	-0.04	0.23^**^	0.19^*^	0.23^*^	0.21^**^	0.20^*^
Takes initiative	0.08	-0.01	0.10	0.03	0.00	0.04	0.09	0.01	0.20^*^
Engaged with peers	-0.26^**^	-0.17^**^	-0.05	-0.32^**^	0.12	0.50^**^	0.02	0.27^**^	0.12
Reflects on development	-0.06	0.03	-0.04	-0.10	0.12	0.34^**^	-0.11	0.04	0.22^**^
Director ratings									
Shows interest	-0.14	-0.12	-0.04	-0.11	0.05	0.08	-0.08	0.06	0.01
Positive attitude	-0.13	-0.14	0.02	-0.16^*^	0.05	0.09	-0.05	0.12	-0.05
Arrives prepared	-0.13	-0.12	-0.13	0.03	0.21^*^	0.10	0.09	0.12	0.09
Takes initiative	-0.08	-0.05	-0.10	0.01	-0.05	0.04	0.08	0.14	0.07
Engaged with peers	-0.31^**^	-0.31^**^	-0.08	-0.26^*^	-0.21^*^	-0.03	-0.13	0.03	-0.12
Reflects on development	-0.19^**^	-0.16^**^	-0.09	-0.11	0.08	0.11	0.08	0.15^*^	0.08
Leadership potential	-0.05	-0.03	-0.05	-0.02	0.04	-0.04	-0.02	0.02	-0.09
Derailment potential	-00.07	0.07	-0.02	-0.18^*^	0.22^**^	0.12	0.03	0.00	0.14

*^∗^p < 0.05, ^∗∗^p < 0.01, one-tailed. n = 113-123. Narcissism sub-scales: LA (Leadership/Authority), GE (Grandiose/Exhibitionism), and EE (Entitlement/Exploitativeness). Big 5 personality characteristics: E (Extraversion), A (Agreeableness), C (Conscientiousness), ES (Emotional Stability), and II (Intellectual Imagination).*

Total NPI was also correlated with several of the self-ratings of LDEB items, including showing interest in areas outside of their field (*r* = -0.25, *p* < 0.01), displaying a positive attitude throughout events (*r* = -0.16, *p* < 0.01), and engaging with peers (*r* = -0.26, *p* < 0.01). Total NPI was also negatively correlated with director ratings of engagement with peers (*r* = -0.31, *p* < 0.01) and reflection on development (*r* = -0.19, *p* < 0.01) during the LDP.

## Discussion

The data support that some personality factors predict behaviors displayed during LDPs. While the results only partially supported the hypotheses, the results showed that narcissism predicts self-ratings and director ratings of LDEBs, especially in relation to engagement with peers and reflection on feedback. Given the importance of these variables and the dearth of research in this area, these findings make an important contribution to our understanding of leader development. To the authors’ knowledge, it is the first study to examine the behaviors of individuals enrolled in leader development.

### Big 5 Personality Outcomes

Extraversion, agreeableness, intellectual curiosity, and emotional stability were positively correlated with overall self-ratings of leader development behavior and several of the specific self-rated leader development behaviors. It is not surprising that conscientiousness predicted self-ratings of “arriving prepared” for leader development events, but it is surprising that conscientiousness was not related to other self-rated and director-rated LDEBs.

Also surprising is the absence of many significant correlations between Big 5 personality characteristics and director ratings of LDP behaviors, especially since in the general leadership literature dimensions of the Big 5, especially conscientiousness, are fairly robust predictors of leadership effectiveness and emergence (Judge et al., 2002). By contrast, agreeableness is not typically a predictor of leadership effectiveness or emergence (Judge et al., 2002), but in this study agreeableness was the most robust of all the personality factors in predicting self-ratings of leader development behavior. It could be that while agreeableness does not predict leader emergence and effectiveness, agreeableness predicts the desire to exhibit team-like behaviors that are necessary to develop strong cohorts in leader development programs. It is also possible that agreeableness predicts behaviors that are seen as positive in leader development programs (e.g., showing interest in all subjects; engaging with peers without being competitive), even if these same behaviors do not necessarily predict leader emergence. If so, it could be that agreeableness is particularly relevant when organizations hope to use leadership development to increase social capital within the firm (Galli and Müller-Stewens, 2012). In this way, it is possible that factors that predict effective leadership development are different from factors that predict effective leader performance. Still, agreeableness did not predict overall director ratings of LDEBs nor did agreeableness predict any of the director ratings of specific LDEBs.

There are a number of explanations for the contrast between self-ratings and director ratings of LDEBs and the correlations with Big 5 personality characteristics. First, and most obvious, is that the positive correlations reflect common rater bias. However, the narcissism total and subscales were generally correlated with leader development ratings in the expected direction, giving some validity to the quality of the personality data and the self-report measures of leader development behavior. A second potential explanation is that personality predicts self-ratings of how one believes a person should behave when enrolled in a leader development program. Thus, the positive and significant correlation between agreeableness and the leader development behaviors reflects less how the highly agreeable person actually behaves during leader development, but rather how the highly agreeable person believes one *should* behave during leader development. Given that the correlation between overall self-rating and overall director rating of leader development behavior failed to reach statistical significance, there is basis for the argument that self-ratings were not an accurate depiction of the director ratings of performance during the program.

### Narcissism Outcomes

After accounting for variance from Big 5 and NPI dimensions, total NPI still accounted for significant variance in self-ratings and director ratings of LDEBs. Overall, these results are important given that narcissists tend to be attracted to positions of power (Kets de Vries and Miller, 1985), often emerge as leaders (Paulhus, 1998; Brunell et al., 2008; Judge et al., 2009; Wales et al., 2013), and are often prescribed leader development as an intervention for problematic behavior (Harms et al., 2011). This is the first study that indicates that narcissists might be particularly ill-suited for leader development programs. The contrast between this study and the Harms et al. (2011) study is noteworthy. That is, Harms and colleagues found that narcissism was positively related to some leader development outcomes. In future research, it would be enlightening to have objective measures of leader development behaviors (e.g., observer ratings of interactions; analysis of goals submitted during leader development) in order to better understand the behaviors displayed by narcissists during leader development.

We “unpeeled the onion” by examining specific behaviors displayed as part of an LDP. Total NPI was negatively correlated with director ratings of reflection on development. These findings are particularly meaningful given the importance of reflection on development and feedback during the leader development process (Boyce et al., 2010; Day et al., 2014).

Total NPI was also negatively correlated with engagement with peers. This negative correlation could be the most important component of this study, especially as the correlation was significant and negative for both self-ratings and director ratings of engagement with peers. There has been recent interest in LDPs as a means of building social capital within organizations (Galli and Müller-Stewens, 2012). If narcissists do not reflect on development and attend to feedback, then that is bad for the narcissist. However, if narcissists do not engage with peers, that could be especially problematic, especially if the narcissist takes action that actually damages the ability of the cohort of participants to create a strong network. More research is needed to better understand the relationship between narcissism and cohort development during leader development.

### Limitations

The study included a small sample size (29% response rate) and was based on data collected with university undergraduate students; results may have been different given an adult working population (Wolfe and Johnson, 1995; Twenge and Foster, 2010). Furthermore, the researchers gathered data from 12 different programs within the university. Though all the programs were generally described as LDPs, the curriculums for each program differed. The researchers faced a challenge by not having access to other specific demographic and program-specific information. That is, because the sample was small, and because it was possible that the researchers could identify an individual given the right demographic information, there was only a limited amount of demographic information connected to the data. Analysis could only be completed based on gender in fear that revealing other potentially less common information could make a participant identifiable to peers (e.g., age, race, and religion). However, it is possible that controlling for other demographic variables would have provided important insights (Foster et al., 2003; Twenge and Campbell, 2008; Twenge and Foster, 2010). Furthermore, this study focused on individuals enrolled in LDPs; there was not a comparison group of individuals enrolled in other type of clubs not intended for the purpose of leader development. Finally, the study was based on self-report data which presents limitations (Fisher, 1993).

### Future Research Directions

Future research directions include gathering similar data with seasoned executives participating in leader development programs within organizations. It would ultimately be ideal to examine individual predictors (personality, intelligence), behaviors while enrolled in leader development (engagement, attention to feedback), and outcomes, especially over time, and then compare these behaviors to outcomes. Much money is invested in leader development (Leow and O’Leonard, 2012), yet little is known about the factors related to successful engagement in leader development. Based on these results, it would also be imperative to further understand the role that narcissism plays in the cohort of a leader development program. Rather than looking at the impact of narcissism on individual behaviors, it would be particularly interesting to determine whether having a narcissistic team member impacts the team climate of the cohort or outcomes related to social capital within the organization (Galli and Müller-Stewens, 2012).

It would also be interesting to study the mechanisms that cause narcissism to have a negative impact on director perceptions of engagement. Does the narcissist display more counterproductive work behaviors (Campbell et al., 2011; Spector, 2011), leading the director to develop an overall negative impression of the student, and thus create a negative halo in ratings of other behaviors? Or, do narcissistic students actually engage less and spend little time in reflection? While it would be difficult to gather an objective measure on these behaviors, further research comparing director, peer, and self-ratings, especially using a larger sample, would be interesting. It would also be interesting to explore these relationships using more complex methods of collecting and analyzing personality data (e.g., Sartori et al., 2017) or potentially incorporating other personality indicators such as grit, honesty, and humility into the understanding of behaviors displayed during leader development (Ceschi et al., 2016).

### Practical Implications

The implications of these results suggest that at a university level, program directors should prescreen for narcissism in the application process. To do this, directors might use a narcissism measure to directly measure the applicants’ narcissistic tendencies. As a proxy, directors might ask questions referring to past experiences with groups, learning something they were not initially interested in, or interest in self-development. Such questions might be helpful for detecting negative behaviors often associated with narcissism. Still, it should be noted that just as in organizational human resource systems, prescreening for narcissism has its challenges (Campbell et al., 2011). When highly narcissistic individuals are included in a program, it having awareness of how these behaviors could impact the individual’s development and the development of the group could alleviate some of the potential negative outcomes.

## Conclusion

Organizational leaders note leader development as a top human capital concern for organizations. Yet there is still much that we do not understand about building effective leader development programs, especially concerning the role individual differences play in predicting LDEBs. This study contributes to that area of the literature. Specifically, Big 5 personality factors predict somewhat consistently self-ratings of leader development behavior. More importantly, narcissism is negatively related to self-ratings and director ratings of leader development behavior. If substantiated in future research, these findings could have tremendous impact on decisions regarding the role of individual differences in the selection process for LDPs.

## Author Contributions

All three authors contributed to the paper in providing substantial contributions to the conception or design of the work, drafting the work, and revising it critically for important intellectual content, final approval of the version to be published, and agreement to be accountable for all aspects of the work in ensuring that questions related to the accuracy or integrity of any part of the work are appropriately investigated and resolved.

## Conflict of Interest Statement

The authors declare that the research was conducted in the absence of any commercial or financial relationships that could be construed as a potential conflict of interest.

## APPENDIX A

### Director Questionnaire

*Note.* Students were asked the first six questions worded appropriately for self-report.

Please answer the following question based on your perception of each student’s performance.

1. While attending meetings and events, he/she shows interest for all subjects, even those not directly related to his/her interests.
2. While attending meetings and events, he/she maintains a positive attitude and demeanor.
3. He/she arrives prepared for events, able to make meaningful comments and ask well-informed questions.
4. When this student faces a problem, he/she takes the initiative to solve the problem before concerning others, including the director.
5. This student is engaged with his/her peers in a way that benefits all involved. He/she shows concern for other students’ development without being unnecessarily competitive.
6. This student takes time to reflect on his/her development in this program.
7. Do you think this student has a high potential to be successful as a leader?
8. Does this student have a high potential for derailing^∗^ during his/her career?
  a. Definition of “to derail”: to obstruct the progress of; to upset the stability or composure of.

## References

[B1] American Psychiatric Association (2013). *Diagnostic and Statistical Manual of Mental Disorders*, 5th Edn. Arlington, TX: American Psychiatric Publishing 10.1176/appi.books.9780890425596

[B2] Avolio B. J. (2005). *Leadership Development in Balance: MADE/Born*. Mahwah, NJ: Lawrence Erlbaum Associates, Inc 10.4324/9781410611819

[B3] Barrick M. R. Mount M. K. (1991). The big five personality dimensions and job performance: a meta-analysis. *Pers. Psychol.* 44 1–26. 10.1111/j.1744-6570.1991.tb00688.x

[B4] Bartlett M. S. (1954). A note on the multiplying factors for various chi square approximations. *J. R. Stat. Soc.* 16 296–298.

[B5] Bell S. (2007). Deep-level composition variables as predictors of team performance: a meta-analysis. *J. Appl. Psychol.* 92 595–613. 10.1037/0021-9010.92.3.595 17484544

[B6] Blair C. A. Helland K. Walton W. (2017). Leaders behaving badly: the relationship between narcissism and unethical leadership behavior. *Leadersh. Organ. Dev. J.* 38 333–346. 10.1108/LODJ-09-2015-0209

[B7] Blair C. A. Hoffman B. J. Helland K. (2008). Narcissism and manager effectiveness: An empirical examination of the dark side. *Hum. Perform.* 21 254–276. 10.1080/08959280802137705

[B8] Boyce L. A. Zaccaro S. J. Wisecarver M. Z. (2010). Propensity for self-development of leadership attributes: understanding, predicting, and supporting performance of leader self-development. *Leadersh. Q.* 21 159–178. 10.1016/j.leaqua.2009.10.012

[B9] Brailovskaia J. Bierhoff H.-W. Margraf J. (2017). How to identify Narcissism with 13 Items? Validation of the German narcissistic personality inventory-13 (G-NPI-13). *Assessment* 10.1177/1073191117740625 [Epub ahead of print]. 29117708

[B10] Brunell A. B. Gentry W. A. Campbell W. K. Hoffman B. J. Kuhnert K. W. DeMarree K. G. (2008). Leader emergence: the case of the narcissistic leader. *Pers. Soc. Psychol. Bull.* 34 1663–1676. 10.1177/0146167208324101 18794326

[B11] Campbell W. K. Hoffman B. J. Campbell S. M. Marchisio G. (2011). Narcissism in organizational contexts. *Hum. Resour. Manag. Rev.* 21 268–284.

[B12] Ceschi A. Sartori R. Dickert S. Costantini A. (2016). Grit or honesty-humility? New insights into the moderating role of personality between the health impairment process and counterproductive work behavior. *Front. Psychol.* 7:1799. 10.3389/fpsyg.2016.01799 28018250 PMC5147463

[B13] Collins D. Holton E. (2004). The effectiveness of managerial leadership development programs: a meta-analysis of studies from 1982 to 2001. *Hum. Resour. Dev. Q.* 15 217–248. 10.1002/hrdq.1099

[B14] Conger J. (1990). The dark side of leadership. *Organ. Dyn.* 19 44–55. 10.1016/0090-2616(90)90070-6

[B15] Costa P. T. Jr. McCrae R. R. (1988). Personality in adulthood: a six-year longitudinal study of self-reports and spouse ratings on the NEO personality inventory. *J. Pers. Soc. Psychol.* 54 853–863. 10.1037/0022-3514.54.5.853 3379583

[B16] Day D. V. (2000). Leadership development: a review in context. *Leadersh. Q.* 11 581–613. 10.1016/S1048-9843(00)00061-8

[B17] Day D. V. Fleenor J. W. Atwater L. E. Sturm R. E. McKee R. A. (2014). Advances in leader and leadership development: a review of 25 years of research and theory. *Leadersh. Q.* 25 63–82. 10.1016/j.leaqua.2013.11.004

[B18] Day D. V. Halpin S. M. (2004). “Growing leaders for tomorrow: An introduction,” in *Leader Development for Transforming Organizations: Growing Leaders for Tomorrow*, eds Day D. V. Zaccaro S. J. Halpin S. M. (Mahwah, NJ: Lawrence Erlbaum Associates Publishers), 3–22. 10.4324/9781410610102

[B19] Day D. V. Harrison M. M. Halpin S. M. (2012). *An Integrative Approach to Leader Development: Connecting Adult Development, Identity, and Expertise*. New York, NY: Routledge 10.4324/9780203809525

[B20] Digman J. M. (1989). Five robust trait dimensions: development, stability, and utility. *J. Pers.* 57 195–214. 10.1111/j.1467-6494.1989.tb00480.x 2671337

[B21] Fisher R. J. (1993). “Social desirability bias and the validity of indirect questioning“. *J. Consum. Res.* 20 303–315. 10.1086/209351

[B22] Foster J. D. Campbell W. K. Twenge J. M. (2003). Individual differences in narcissism: inflated self-views across the lifespan and around the world. *J. Res. Pers.* 37 469–486. 10.1016/S0092-6566(03)00026-6

[B23] Funderburg S. A. Levy P. E. (1997). The influence of individual and contextual variables on 360-degree feedback system attitudes. *Group Organ. Manag.* 22 210–235. 10.1177/1059601197222005 19709012

[B24] Galli E. B. Müller-Stewens G. (2012). How to build social capital with leadership development: lessons from an explorative case study of a multibusiness firm. *Leadersh. Q.* 23 176–201. 10.1016/j.leaqua.2011.11.014

[B25] Gentile B. Miller J. D. Hoffman B. J. Reidy D. E. Zeichner A. Campbell W. K. (2013). A test of two brief measures of grandiose narcissism: the narcissistic personality inventory-13 and the narcissistic personality inventory-16. *Psychol. Assess.* 25 1120–1136. 10.1037/a0033192 23815119

[B26] Goldberg L. R. (1990). An alternative “description of personality”: the big-five factor structure. *J. Pers. Soc. Psychol.* 59 1216–1229. 10.1037/0022-3514.59.6.1216 2283588

[B27] Goldberg L. R. (1992). The development of markers for the Big-Five factor structure. *Psychol. Assess.* 4 26–42. 10.1037/1040-3590.4.1.26 11575511

[B28] Hannah S. T. Avolio B. J. (2010). Ready or not: how do we accelerate the developmental readiness of leaders? *J. Organ. Behav.* 31 1181–1187. 10.1002/job.675

[B29] Hannum K. M. Craig S. B. (2010). Introduction to special issue on leadership development evaluation. *Leadersh. Q.* 21 581–582. 10.1016/j.leaqua.2010.06.001

[B30] Harms P. D. Spain S. M. Hannah S. T. (2011). Leader development and the dark side of personality. *Leadersh. Q.* 22 495–509. 10.1037/a0035790 24490967

[B31] Hogan R. Hogan J. (2001). Assessing leadership: a view from the dark side. *Int. J. Select. Assess.* 9 40–51. 10.1111/1468-2389.00162

[B32] Judge T. A. Bono J. E. Ilies R. Gerhardt M. W. (2002). Personality and leadership: a qualitative and quantitative review. *J. Appl. Psychol.* 87 765–780. 10.1037/0021-9010.87.4.765 12184579

[B33] Judge T. A. Piccolo R. F. Kosalka T. (2009). The bright and dark side of leader traits: A review and theoretical extension of the leader trait paradigm. *Leadersh. Q.* 20 855–875. 10.1016/j.leaqua.2009.09.004

[B34] Kaiser H. (1970). A second generation Little Jiffy. *Psychometrika* 35 401–415. 10.1007/BF02291817

[B35] Kaiser H. (1974). An index of factorial simplicity. *Psychometrika* 39 31–36. 10.1007/BF02291575

[B36] Kaiser R. B. Curphy G. (2013). Leadership development: the failure of an industry and the opportunity for consulting psychologists. *Consult. Psychol. J.* 65 294–302. 10.1037/a0035460

[B37] Kearney K. S. (2010). Grappling with the gods: reflections for coaches of the narcissistic leader. *Int. J. Evid. Based Coach. Mentor.* 8 1–13.

[B38] Kernis M. H. Sun C. (1994). Narcissism and reactions to interpersonal feedback. *J. Res. Pers.* 28 4–13. 10.1006/jrpe.1994.1002

[B39] Kets de Vries M. F. R. Miller D. (1985). Narcissism and leadership: an object relations perspective. *Hum. Relat.* 38 583–601. 10.1177/001872678503800606 9661312

[B40] Kets de Vries M. F. R. (2004). Putting leaders on the couch: a conversation with Manfred F. F. Kets de Vries. Interview by Diane L. Coutu. *Harv. Bus. Rev.* 83 64–71. 14723178

[B41] Leow L. O’Leonard K. (2012). *Leadership Development Factbook 2012: Benchmarks and Trends in US Leadership Development*. Available at: www.bersin.com/library.

[B42] Lubit R. (2002). The long-term organizational impact of destructively narcissistic managers. *Acad. Manag. Execut.* 16 127–138.

[B43] Maccoby M. (2004). Narcissistic leaders: the incredible pros, the inevitable cons. *Harv. Bus. Rev.* 82 92–101.

[B44] Maurer T. J. Mitchell D. R. D. Barbeite F. G. (2002). Predictors of attitudes toward a 360-degree feedback system and involvement in post-feedback management development activity. *J. Occup. Organ. Psychol.* 75 87–107. 10.1348/096317902167667

[B45] Maurer T. J. Tarulli B. A. (1994). Investigation of perceived environment, perceived outcome, and person variables in relationship to voluntary development activity by employees. *J. Appl. Psychol.* 79 3–14. 10.1037/0021-9010.79.1.3 8200872

[B46] McCauley C. (2008). *Leader Development: A Review of Research.* Available at: https://www.researchgate.net/publication/266495919_Leader_Development_AReview_of_Research

[B47] Meier L. L. Semmer N. K. (2012). Lack of reciprocity and strain: narcissism as a moderator of the association between feeling under-benefited and irritation. *Work Stress* 26 56–67. 10.1080/02678373.2012.657038

[B48] Noe K. A. Wilk S. L. (1993). Investigation of the factors that influence employees’ participation in development activities. *J. Appl. Psychol.* 78 291–302. 10.1037/0021-9010.78.2.291

[B49] Paulhus D. L. (1998). Interpersonal and intrapsychic adaptiveness of trait self-enhancement: a mixed blessing. *J. Pers. Soc. Psychol.* 74 1197–1208. 10.1037/0022-3514.74.5.1197 9599439

[B50] Penney L. M. Spector P. E. (2002). Narcissism and counterproductive work behavior: Do bigger egos mean bigger problems? *Int. J. Select. Assess.* 10 126–134. 10.1111/1468-2389.00199

[B51] Raskin R. Terry H. (1988). A principle-components analysis of the Narcissistic Personality Inventory and further evidence of its construct validity. *J. Pers. Soc. Psychol.* 54 890–902. 10.1037/0022-3514.54.5.890 3379585

[B52] Rhodewalt F. Morf C. C. (1998). On self-aggrandizement and anger: a temporal analysis of narcissism and affective reactions to success and failure. *J. Pers. Soc. Psychol.* 74 672–685. 10.1037/0022-3514.74.3.672 9523411

[B53] Riggio R. E. (2008). Leadership development: the current state and future expectations. *Consult. Psychol. J.* 60 383–392. 10.1037/1065-9293.60.4.383

[B54] Rosenthal S. A. (2012). *National Leadership Index 2011: A National Study of Confidence in Leadership*. Cambridge, MA: Harvard University.

[B55] Sartori R. Costantini A. Ceschi A. Scalco A. (2017). Not only correlations: a different approach for investigating the relationship between the Big Five personality traits and job performance based on workers and employees’ perception. *Qual. Quant.* 51 2507–2519. 10.1007/s11135-016-0406-2

[B56] Schwartz J. Bersin J. Pelster B. (2014). *Global Human Capital Trends 2014: Engaging the 21-century Workforce*. Westlake, TX: Deloitte University Press.

[B57] Spector P. E. (2011). The relationship of personality to counterproductive work behavior (CWB): An integration of perspectives. *Hum. Resour. Manag. Rev.* 21 342–352. 10.1016/j.hrmr.2010.10.002

[B58] Stewart L. Palmer S. Wilkin H. Kerrin M. (2008). The influence of character: Does personality impact coaching success? *Int. J. Evid. Based Coach. Mentor.* 6 32–42.

[B59] Strang S. E. Kuhnert K. W. (2009). Personality and leadership developmental levels as predictors of leader performance. *Leadersh. Q.* 16 419–439. 10.1016/j.leaqua.2009.03.009

[B60] The Conference Board and McKinsey (2012). *The State of Human Capital 2012 – False Summit: Why the Human Capital Function Still Has Far to Go*. Available at: www.mckinsey.com?$\sim$/media/mckinsey

[B61] Twenge J. M. Campbell S. M. (2008). Generational differences in psychological traits and their impact on the workplace. *J. Manag. Psychol.* 23 862–877. 10.1108/02683940810904367

[B62] Twenge J. M. Foster J. D. (2010). Birth cohort increases in narcissistic personality traits among American college students, 1982-2009. *Soc. Psychol. Pers. Sci.* 1 99–106. 10.1177/1948550609355719

[B63] Wales W. J. Patel P. C. Lumpkin G. T. (2013). In pursuit of greatness: CEO narcissism, entrepreneurial orientation, and firm performance variance. *J. Manag. Stud.* 50 1041–1069. 10.1111/joms.12034

[B64] Wolfe R. N. Johnson S. D. (1995). Personality as a predictor of college performance. *Educ. Psychol. Meas.* 55 177–185. 10.1177/0013164495055002002

