# Supplementary material for: Do Big 5 Personality Characteristics and Narcissism Predict Engagement in Leader Development?
Source: Front Psychol. 2018 Sep 28;9:1817. doi: 10.3389/fpsyg.2018.01817 (PMC6172568; doi:10.3389/fpsyg.2018.01817)
Supplement: Supplementary file 1 [file Table_1.DOCX]

**Table.** Principal component analysis for Leadership Development Performance (LDP) Self and Director ratings

|  | LDP Self Rating | LDP Director Rating |
| --- | --- | --- |
| Kaiser-Meyer-Olin value  Bartlett’s Test of Sphericity  % variance accounted for: | 0.779  186.097*** | 0.860  391.230*** |
| Component 1 | 46.096 | 63.707 |
| Component 2 | 18.150 | 12.690 |
| Component 3 | 12.488 | 8.902 |
| Component 4 | 9.348 | 6.157 |
| Component 5 | 8.297 | 5.048 |
| Component 6 | 5.622 | 3.497 |

*Note.* Significance Bartlett’s test * *p* < .10, ***p* < .05, *** *p* < .01

Source: Authors Dataset

**Factor Loadings**

| LDP Self (Question) | Factor Loading |
| --- | --- |
| 1 | 0.818 |
| 2 | 0.821 |
| 3 | 0.656 |
| 4 | 0.247 |
| 5 | 0.692 |
| 6 | 0.673 |

| LDP Director (Question) | Factor Loading |
| --- | --- |
| 1 | 0.870 |
| 2 | 0.863 |
| 3 | 0.786 |
| 4 | 0.660 |
| 5 | 0.757 |
| 6 | 0.832 |
